# Chloroquine, the Coronavirus Crisis, and Neurodegeneration: A Perspective

**DOI:** 10.3389/fneur.2020.596528

**Published:** 2020-11-13

**Authors:** Giona Pedrioli, Rickie Patani, Paolo Paganetti

**Affiliations:** ^1^Neurodegeneration Research Group, Laboratory for Biomedical Neurosciences, Neurocenter of Southern Switzerland, Ente Ospedaliero Cantonale, Torricella-Taverne, Switzerland; ^2^International PhD Program of the Biozentrum, University of Basel, Basel, Switzerland; ^3^Department of Neuromuscular Disease, UCL Queen Square Institute of Neurology, London, United Kingdom; ^4^The Francis Crick Institute, London, United Kingdom; ^5^Faculty of Biomedical Sciences, Università della Svizzera Italiana, Lugano, Switzerland

**Keywords:** chloroquine, COVID-19, coronavirus, neurodegeneration, clinical trials

## Abstract

On the verge of the ongoing coronavirus pandemic, *in vitro* data suggested that chloroquine, and its analog hydroxychloroquine, may be useful in controlling SARS-CoV-2 infection. Efforts are ongoing in order to test this hypothesis in clinical trials. Some studies demonstrated no evidence of efficacy, whereas in some cases results were retracted after reporting. Despite the lack of scientific validation, support for the use of these compounds continues from various influencers. At the cellular level, the lysosomotropic drug chloroquine accumulates in acidic organelles where it acts as an alkalizing agent with possible downstream effects on several cellular pathways. In this perspective, we discuss a possible modulatory role of these drugs in two shared features of neurodegenerative diseases, the cellular accumulation of aberrantly folded proteins and the contribution of neuroinflammation in this pathogenic process. Certainly, the decision on the use of chloroquine must be determined by its efficacy in the specific clinical situation. However, at an unprecedented time of a potential widespread use of chloroquine, we seek to raise awareness of its potential impact in ongoing clinical trials evaluating disease-modifying therapies in neurodegeneration.

## Introduction

On February 4th, 2020, at the verge of a new pandemic crisis, the anti-malarial drug chloroquine (CQ), was proposed to be highly effective in controlling SARS-CoV-2 infection *in vitro* (1). Soon after, in March 2020, the lack of specific treatments for the rising coronavirus burden induced the U. S. Food and Drug Administration (FDA) to issue an emergency use authorization (EUA) for CQ, and its (more soluble and less toxic) analog hydroxychloroquine (HCQ), as treatments for the control of SARS-CoV-2, the severe acute respiratory syndrome caused by the new coronavirus (2). On June 2020, in light of recent scientific data and analysis, the FDA revoked the EUA for CQ/HCQ, as reported side effects “no longer outweigh the known and potential risks for the authorized use” (3). The most worrisome adverse effects, also listed in the drug labels, include heart rhythm interference related to long QT syndrome, ventricular tachycardia and fibrillation, in particular in combination with QT-prolonging drugs or pre-existing kidney or heart disorders (4–6). Likely differences in dosing regimens when using CQ/HCQ for their approved indications, which are unlikely to meet the concentrations affecting SARS-CoV-2 activity *in vitro*, may explain why the occurrence of these symptoms is uncommon in the medical practice. For instance, while safety profile of CQ in the treatment of rheumatic diseases have been reported up to 500 mg (once daily) (7), in a SARS-CoV-2 clinical trial assessing CQ efficacy a QT interval alteration was observed in patients treated with a higher dose (600 mg, twice daily) (4).

The EUA permission and revocation of the use of CQ/HCQ has caused a stir in the scientific community and beyond during this unstable and delicate pandemic situation. While we acknowledge the natural tendency to dismiss uncomfortable facts and the keenness to move away from CQ, reflecting on possible short and long-term neurological side-effects caused by its use are worthy of a more comprehensive scientific consideration. In particular considering that CQ was, and still is, used as a putative off-label drug to treat SARS-CoV-2, highlights that the response to this pandemic has not always been ruled by a rational and scientific approach. Nonetheless, the possible consequences of using CQ should instigate discussion and warrant a more cautious approach if a similar situation should arise in the future. Here we provide a perspective on the potential interaction of CQ and the neuronal dyshomeostasis observed in common degenerative disorders such as Alzheimer's or Parkinson's disease. We consider the pharmacodynamics and pharmacokinetic attributes of CQ, and its potential effects on the nervous and immune systems.

## Impairment of the Autophagy-Lysosome Pathway

Historically recognized for its undeniable utility in malarial prophylaxis and treatment (8), CQ is also extensively used as a cell biology research compound based on its potent inhibitory activity on autophagic and lysosomal clearance functions. The lipophilic nature of CQ enables a rapid penetration across lipid bilayer membranes. Within the cell, CQ behaves as a lysosomotropic agent, i.e., it undergoes a protonation-based trapping when it reaches the acidic environment present in the lumen of organelles such as lysosomes. Its weak base characteristics results in its accumulation as a function of the pH gradient, the neutralization of the low pH, the inhibition of acidic hydrolases and the impairment of organelle maturation (9). This has led to defining the mode of action of CQ as an inhibitor of both enzymatic activity and organelle fusion resulting in halting autophagy flux and endo-lysosomal degradative function (10).

## Impairment of the Proteasome System

Beside the autophagy-lysosome pathway, experimental evidence proposes that CQ is a weak antagonist of the proteasome system, causing accumulation of ubiquitinated proteins in mammalian cells (11, 12). Mechanistically, CQ acts as an allosteric inhibitor of the enzymatic activity of the 26S proteasome degradation system (13). Together, these studies highlight a likely dual inhibitory effect of CQ in the two major metabolic systems regulating cellular proteostasis. Moreover, the presence of CQ modify the heat-shock response regulating protein chaperons expression in mammalian cells (14) with additional consequences on the mammalian proteostasis and on the drug resistance of the malaria parasite *Plasmodium* (15).

## Access to the Central Nervous System: A Pharmacokinetic Perspective

CQ can be administered orally as a phosphate salt and it is efficiently absorbed by the upper intestinal tract, thus permitting a high drug bioavailability. Plasma CQ concentration peaks at 8–12 h post-administration. CQ is slowly metabolized mainly in the liver by cytochrome P450 enzymes and is converted into desethylchloroquine. Further desethylation leads to the second, less frequent, metabolite bisdesethylchloroquine. CQ and its active metabolites have a remarkably slow elimination rate, which in turns may facilitate a widespread tissue exposure, indeed reflected in a large distribution volume. Although about 70% of CQ is directly cleared by the kidneys, CQ and its metabolites are detected in blood plasma for as long as 70 days, and in the urine up to 1 year post-administration. Notably, the equally active CQ enantiomers differ in their overall elimination kinetics. In animals, the concentration of CQ reaches 10-to-700 times higher levels in the liver, spleen, kidney, and lung when taking that detected in the plasma as reference (16, 17). Despite some controversy around the efficacy of CQ to penetrate the blood-brain barrier (BBB), animal studies demonstrate that this drug and its analogs can penetrate and reach a concentration that is sufficient to exert its effects within the central nervous system (CNS) (18–20). Nonetheless, reported neurological side effects of CQ and its analogs implicate a non-yet fully confirmed CNS exposure in humans (21). In particular, CQ/HCQ can have potential adverse neuropsychiatric effects, similar to symptoms occurring in neurodegenerative disorders, such as agitation, emotional instability, anxiety, irritability and, rarely, psychosis (22, 23).

Therefore, at a time were CQ is used in clinical trials or as a self-remedy, and as long it is not excluded that the CNS is a target tissue of the drug, predicting possible consequences of CQ exposure in the brain is important in order to prevent possible neurological effects, e.g., for patients affected by neurodegenerative disorders.

## Modulation of Autophagy

**Figure 1:**
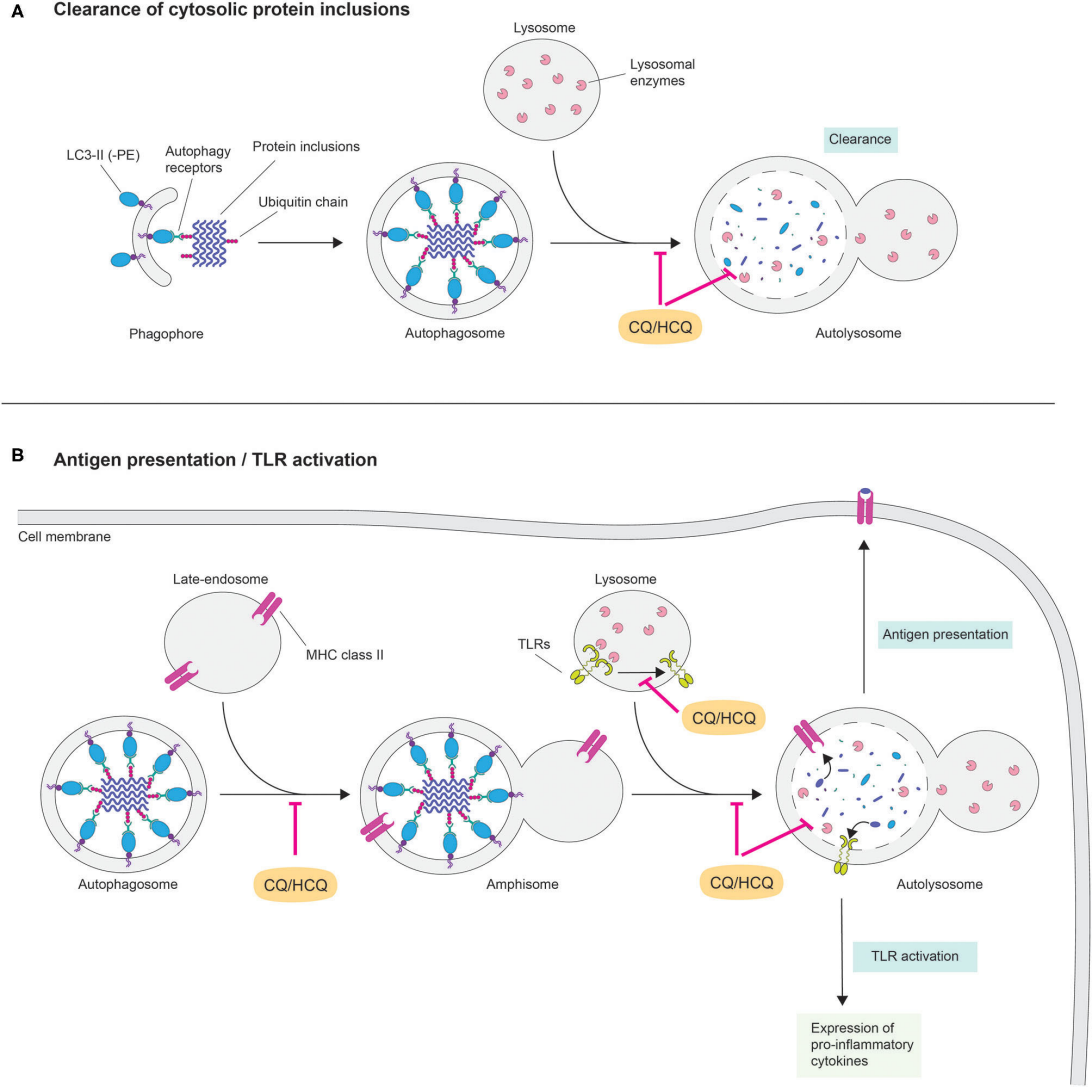
Potential cellular and molecular mechanisms of chloroquine in neurodegeneration. The lysosomotropic agent chloroquine (CQ) rapidly penetrates across lipid bilayer membranes and following a pH gradient accumulates within lysosomes. In these acidic organelles, CQ behaves as a weak base by increasing the pH, which in turns affects the activity of lysosomal hydrolases. Disruption of lysosomal activity prevents interaction and fusion among organelles of the autophagy-lysosome and of the endocytic pathways. This cellular condition may have dichotomic effects in the pathogenesis of neurodegenerative diseases by **(A)** inhibiting cytosolic clearance of aberrantly protein fibrils and **(B)** preventing MHC class II-mediated antigen presentation and preventing the expression of pro-inflammatory cytokines *via* TLR activation.

Although little is known regarding the direct effects of CQ on the CNS, the latter is particularly vulnerable to disruptions of the cellular degradative pathways. Indeed, terminally differentiated neurons rely on efficient quality control systems such as the autophagic-lysosomal pathway for maintaining their delicate proteostasis, which is gradually impaired as the brain ages (24). Autophagy is responsible for delivering cytoplasmic material to the lysosome for degradation. Autophagy is subdivided in three distinct processes that differ in their mechanism of recognition and delivery of substrates to lysosomes: chaperon mediated autophagy (CMA), macroautophagy and microautophagy (25). The selective clearance of aberrant proteins is primarily carried out by CMA and macroautophagy. In CMA, proteins that bear a pentapeptide degradation signal (KFERQ-like) are recognized by the chaperone heat-shock cognate 70 (HSC70) and delivered through the CMA adaptor lysosomal membrane associated protein 2A (LAMP2A) to the lysosomal lumen for degradation. In contrast, aberrantly folded proteins that are prone to self-aggregate into β-sheet-rich oligomers and higher order aggregates are sequestered by macroautophagy together with small portion of the cytoplasm. These substrates are encapsulated within an intermediate double lipid bilayer membrane organelle termed “autophagosome” and directed toward lysosomes, where upon membrane fusion, cargos are liberated in the hydrolases-enriched lysosomal lumen for enzymatic digestion (26) (Figure 1A).

Most late onset neurodegenerative disorders share the progressive deposition of aberrantly folded, β-sheet-rich protein aggregates into ubiquitinated intraneuronal inclusions. Each disorder is characterized by the aggregation of specific proteins: examples are beta-amyloid and TAU in Alzheimer's disease (27), NACP/α-synuclein in Parkinson's disease (28), huntingtin in Huntington's disease (29), TDP-43 in amyotrophic lateral sclerosis and frontotemporal lobar degeneration (30). Nevertheless, another key pathological hallmark of these otherwise clinically and etiologically diverse disorders is the progressive impairment in the autophagy-lysosome degradation pathway. This is exemplified by the fact that mutations of genes regulating autophagy and lysosome activity are associated to the most frequent late-onset forms of neurodegeneration (31). Furthermore, experimental animal models demonstrate that autophagy deficiency accelerates protein aggregation and behavioral phenotypes of neurodegeneration. Evidence that CQ exposure on neurons may lead to a similar outcome are known since long time (32). More recently the activity of CQ on the amyloidogenic processing of the amyloid precursor protein by neurons (33, 34) as well as on huntingtin accumulation in brain (20) were reported. CQ also modulates autophagic flux (35) and mitochondrial homeostasis by an autophagic process (36). CQ is also linked to neuronal death in primary cultures (37, 38). These facts are reinforced by studies demonstrating that autophagy stimulation can clear intra-neuronal insoluble protein inclusions with amelioration of behavioral phenotypes in animal models of neurodegenerative diseases (26) (Table 1). Nevertheless, macroautophagy may also favor seeded propagation of aberrantly folded neurodegeneration-associated TAU mediated by extracellular vesicles (39).

**Table 1:** Examples of evidence for beneficial effect of autophagy stimulation in murine brain.

**Compound**	**Targeted** **pathway**	**Ectopic** **expression**	**Disease** **model**	**Outcome**	**References**
Rapamycin	Mammalian target of rapamycin (mTor)	Human TDP-43	Amyotrophic lateral sclerosis	Reduced TDP-43 inclusions and improved learning/memory impairment	(56)
Rapamycin	mTor	Human APP Human TAU Human PSEN1	Alzheimer's disease	Reduced beta-amyloid and TAU deposition and improved learning defects	(57)
Rapamycin	mTor	Human NACP	Parkinson's disease	Reduced aggregation of NACP and associated pathology	(58)
CCI-779	mTor	Human HTT	Huntington's disease	Reduced huntingtin aggregates formation and improved behavioral phenotype	(59)
Trehalose	mTor-independent	Human SOD1	Amyotrophic lateral sclerosis	Reduced accumulation of SOD1 and enhanced motoneuronal survival	(60)
Trehalose	mTor-independent	Human APP Human PSEN1	Alzheimer's disease	Reduced beta-amyloid plaque deposition and improved learning defects	(61)
Trehalose	mTor-independent	Human TAU	Alzheimer's disease	Reduced TAU inclusions and increased brain neuronal survival	(62)
Trehalose	mTor-independent	Human HTT	Huntington's disease	Reduced formation of polyglutamine aggregates and amelioration of motor dysfunction	(63)
Lithium	Inositol synthesis	Human APP Human PSEN1	Alzheimer's disease	Reduced beta-amyloid plaque formation and improved memory deficits	(64)
Lithium	Inositol synthesis	Human SOD1	Amyotrophic lateral sclerosis	Reduced SOD1 aggregates and increased brain neuronal survival	(65)
Carbamazepine	Inositol synthesis	Human APP Human PSEN1	Alzheimer's disease	Reduced beta-amyloid plaque formation and improved memory deficits	(66)
Carbamazepine	Inositol synthesis	Human TDP-43	Amyotrophic lateral sclerosis	Reduced TDP-43 inclusions and improved learning/memory impairement	(56)
Spermidine	Acetyl transferases synthesis	Human TDP-43	Amyotrophic lateral sclerosis	Reduced TDP-43 inclusions and improved learning/memory impairement	(56)
Verapamil	Ca^2+^ channel	Human SOD1	Amyotrophic lateral sclerosis	Reduced SOD1 aggregates and prolonged animal survival	(67)
Felodipine	Ca^2+^ channel	Human NACP	Parkinson's disease	Reduced aggregation of NACP and improved behavioral phenotype	(68)
Calpastatin	Calpain	Human HTT	Huntington's disease	Reduced HTT aggregates formation and improved locomotor function	(69)
Beclin-1	Beclin-1 dependent	Human NACP	Parkinson's disease	Reduced aggregation of NACP	(70)
LAMP2A	LAMP2A dependent	Human NACP	Parkinson's disease	Reduced generation of aberrant NACP species	(71)

## Modulation of Inflammatory Response

Another increasingly documented feature of neurodegenerative disorders is the chronic inflammation of the CNS (neuroinflammation). Although a causal relationship has not yet been demonstrated, there are studies reporting a correlation between prolonged treatment with non-steroidal anti-inflammatory drugs and decreased risk for Alzheimer's and Parkinson's disease (40, 41). Activation of CNS-resident macrophages (microglia cells) around senile plaques has been documented in transgenic mouse model of Alzheimer's disease (42). These phagocytic cells actively uptake beta-amyloid and acquire an activated phenotype characterized by morphological changes and by an increased production of pro-inflammatory modulators such as the major histocompatibility complex (MHC) class II, several interleukins and tumor necrosis factor alpha. Persistent microglial activation is associated with cellular senescence, neurotoxicity and subsequent disease progression (43). Recent studies suggest that this may also involve deleterious reactive transformation in astrocytes (44). Notably, elimination of senescent glial cells, which are known to release proinflammatory modulators, is beneficial (45–47).

Against this background, HCQ's clinical efficacy in treating autoimmune inflammatory diseases, such as rheumatoid arthritis and systemic lupus erythematosus, is well-documented (48). Current hypotheses in the field are linked to an indirect effect of HCQ in modulating the inflammatory response (Figure 1B). Specifically, interference of lysosomal activity might affect several immunomodulatory pathways. One intuitive mechanism is the inhibition of antigen presentation via the autophagy-lysosome pathway. As lysosomes are the main organelles for hydrolytic processing, they reside at the intersection between different pathways delivering intracellular and extracellular cargos on route to degradation (49). This context provides a unique cellular environment for the binding of antigens to MHC class II. For instance, a recent report suggests that extracellular proteins are hydrolysed in endocytic compartments and delivered to MHC class II-containing lysosomes as antigenic peptides before being presented to CD4^+^ T cells (50). Nevertheless, functional lysosomes are required for antigenic peptide-binding to MHC class II molecules and the alkalizing properties that HCQ exerts in these organelles might impair this process. Another possibility is that HCQ interferes in Toll-like receptor (TLR) signaling. In mammals, TLRs are a group of transmembrane pattern-recognition receptors that initiate innate immune response to infection by sensing pathogen macromolecules. However, TLRs can also be activated in the absence of pathogen infection (51). Indeed, activated microglia surrounding beta-amyloid plaques in Alzheimer's disease brains display up-regulated levels of TLRs (52, 53). A recent report indicates that, in order to be functional, TLR7 requires proteolytic cleavage in lysosomes (48). Thus, interfering with lysosomal pH via lysosomotropic agents may prevent activation of TLRs. Moreover, evidence exists for a mode of action of CQ/HCQ independently of its effect on lysosomal function, as shown for its ability to interference with interleukin-2 production (54). Although the precise mechanism(s), by which CQ/HCQ inhibits inflammatory response, requires further investigation, its potential role in disrupting the integrity of the CNS immune system in neurodegenerative disorders is an intriguing and noteworthy hypothesis. Evidence for a possible role of CQ in modulating inflammation and autophagic death of neurons in the brain exists (55).

## Conclusions

Given the demographic, in particular associated to aging, of people affected by neurodegenerative disorders and patients more vulnerable to develop a serious SARS-CoV-2 disease course, the possibility that CQ, or one of its analogs, will be prescribed/self-consumed by patients enrolled in clinical trials (or outside this context and off license) is worth considering. However, the use of CQ and its analogs must be determined by clinical need, so that prescribing CQ may be opportune and take priority depending on specific clinical context. However, at a time of a potential widespread use of CQ, in order to mitigate the risk of potential misinterpretation in ongoing clinical trials evaluating disease-modifying therapies in neurodegeneration, we seek to raise awareness and caution that the use of CQ and its analogs needs to be clearly documented and carefully considered in interpreting trial outcomes in this arena and beyond.

## Data Availability Statement

The original contributions presented in the study are included in the article/supplementary material, further inquiries can be directed to the corresponding author.

## Author Contributions

All authors listed have made a substantial, direct and intellectual contribution to the work, and approved it for publication.

## Conflict of Interest

The authors declare that the research was conducted in the absence of any commercial or financial relationships that could be construed as a potential conflict of interest.

## Abbreviations

CQ: Chloroquine
FDA: U. S. Food and Drug Administration
EUA: Emergency use authorization
CNS: Central nervous system
CMA: Chaperon mediated autophagy
HSC70: Chaperone heat-shock cognate 70
LAMP2A: Lysosomal membrane associated protein 2A
MHC: Major histocompatibility complex
TLR: Toll-like receptor.
